# Modeling the Blood-Brain Barrier Permeability of Potential Heterocyclic Drugs via Biomimetic IAM Chromatography Technique Combined with QSAR Methodology

**DOI:** 10.3390/molecules29020287

**Published:** 2024-01-05

**Authors:** Małgorzata Janicka, Małgorzata Sztanke, Krzysztof Sztanke

**Affiliations:** 1 Department of Physical Chemistry, Faculty of Chemistry, Institute of Chemical Science, Maria Curie-Skłodowska University, 20-031 Lublin, Poland; malgorzata.janicka@mail.umcs.pl; 2 Department of Medical Chemistry, Medical University of Lublin, 4A Chodźki Street, 20-093 Lublin, Poland; malgorzata.sztanke@umlub.pl; 3 Laboratory of Bioorganic Compounds Synthesis and Analysis, Medical University of Lublin, 4A Chodźki Street, 20-093 Lublin, Poland

**Keywords:** log BB, QSAR, biomimetic chromatographic technique, IAM column, potential pharmaceuticals, drug-like compounds for drug development

## Abstract

Penetration through the blood-brain barrier (BBB) is desirable in the case of potential pharmaceuticals acting on the central nervous system (CNS), but is undesirable in the case of drug candidates acting on the peripheral nervous system because it may cause CNS side effects. Therefore, modeling of the permeability across the blood-brain barrier (i.e., the logarithm of the brain to blood concentration ratio, log BB) of potential pharmaceuticals should be performed as early as possible in the preclinical phase of drug development. Biomimetic chromatography with immobilized artificial membrane (IAM) and the quantitative structure-activity relationship (QSAR) methodology were successful in modeling the blood-brain barrier permeability of 126 drug candidates, whose experimentally-derived lipophilicity indices and computationally-derived molecular descriptors (such as molecular weight (MW), number of rotatable bonds (NRB), number of hydrogen bond donors (HBD), number of hydrogen bond acceptors (HBA), topological polar surface area (TPSA), and polarizability (α)) varied by class. The QSARs model established by multiple linear regression showed a positive effect of the lipophilicity (log k_w, IAM_) and molecular weight of the compound, and a negative effect of the number of hydrogen bond donors and acceptors, on the log BB values. The model has been cross-validated, and all statistics indicate that it is very good and has high predictive ability. The simplicity of the developed model, and its usefulness in screening studies of novel drug candidates that are able to cross the BBB by passive diffusion, are emphasized.

## 1. Introduction

The possibilities of modern science, including organic synthesis and computational potential, suggest that the search for new bioactive compounds is limited only by human imagination. However, identifying which of the obtainable structures will possess the desired features is much more complicated. What is important is not only the possible therapeutic effects of modeled drug-like compounds, but also the prediction of undesirable side effects of their action. Determining the ADME-Tox profile [1] requires tedious, expensive, and often unethical animal tests, the use of which in screening tests is at least unreasonable. Alternative methods are helpful in this case. Biomimetic chromatographic techniques, known as in vitro methods for testing bioactive compounds for drug development, are extremely helpful in such studies. Combined with in silico techniques, these methods are gaining popularity and recognition in the screening of potentially active new molecules. In recent years, the role of chromatography with immobilized artificial membrane (IAM) in drug discovery has been emphasized, with numerous publications highlighting its applications in predicting the pharmacokinetic profiles of drug-like compounds [2,3,4,5,6,7,8,9,10,11,12,13,14].

Blood-brain barrier (BBB) permeability is one of the most important biological properties to consider during the design and assessment of future potential medicines. It is important both for the development of central nervous system (CNS)-active drugs and for peripherally-selective pharmaceuticals. In the latter case, passage into the central nervous system is undesired because it may lead to CNS side effects. Penetration through the blood-brain barrier of potential drugs should be predicted as early as possible during preclinical studies [15,16]. The most common numeric value describing permeability across the BBB is the log BB parameter [17] defined as the logarithmic ratio between the concentration of a compound in the brain (c_brain_) and the blood (c_blood_) (Equation (1)):

(1) $$\log\;BB=\frac{c_{brain}}{c_{blood}}$$

It is already common and accepted knowledge that lipophilicity (expressed as the logarithmic *n*-octanol/water partition coefficient, log P) is the main property that determines a compound’s penetration through the BBB [18,19,20]. This parameter is present in almost all QSARs models, but the direct measurement of the log P value is a tedious, expensive and very demanding technique, with which indirect techniques such as liquid chromatography, especially biomimetic, compete [2,3,4,5,6,7]. Additionally, in silico methods, although simple and relatively inexpensive, are not without drawbacks. They often lead to different results depending on the software used [21].

A large number of drug candidates (**1**–**126**; classes **I**–**XVI**, Table 1), whose pharmacological and analytical significance is described in the Supplementary material, were used as an important set in our current studies. As these drug-like molecules may be used in future as potential pharmaceuticals, they should be subjected to further in vitro investigation employing the biomimetic chromatographic technique aimed at modeling their permeation through the blood-brain barrier. The results of such studies would be very important in the preclinical phase of drug development.

In the present paper, reversed-phase liquid chromatography with immobilized artificial membrane combined with the quantitative structure-activity relationship (QSAR) methodology was applied with the aim of developing a novel, superior QSAR model—established by multiple linear regression—which would predict the penetration through the blood-brain barrier of a large number of structurally diversified drug-like molecules (126 structures whose physicochemical descriptors differ by class), meeting not only the rule of five but also more stringent requirements. The subsequent purpose was to determine which increased physicochemical and structural features (among them: lipophilicity, molecular weight (MW), number of rotatable bonds (NRB), topological polar surface area (TPSA), polarizability (*α*), number of hydrogen bond donors (HBD), number of hydrogen bond acceptors (HBA)) of the tested compounds favor, and which hinder, penetration of the blood-brain barrier.

## 2. Results and Discussion

### 2.1. In Silico Characteristics

The descriptors of 126 tested heterocyclic molecules calculated in silico on the basis of molecular structures (MW, NRB, HBD, HBA, TPSA, and α values), as presented in Table 2, indicate that they meet the rules relating to the characteristics of drug-related compounds, both Lipiński’s general rule of five (Ro5 [18]) and more detailed guidelines. 

The molar weights of the investigated compounds are in the range of 186.21–430.11 g mol^−1^, which gives an average value of 323.47 g mol^−1^. According to Lipiński’s Ro5 [18], the MW should be less than 500 g mol^−1^. They also meet the more stringent requirements of Ghose et al. [24], according to which MW should be in the range of 160–480 g mol^−1^, and Clark [19], according to which drug candidates require MW ≤ 480 g mol^−1^. 

The topological polar surface areas of the tested compounds are in the range of 33.95–121.85 Å^2^. For good brain permeation, TPSA (which is a measure of a molecule’s hydrogen-bonding area) should be below a certain limit: van de Waterbeemd et al. suggest a limit of 90 Å^2^ [20], whereas Kelder et al. have a lower limit of 60–70 Å^2^ [25]. 

HBD values range from 0 to 3 and HBA from 4 to 10. According to Ro5, HBD should be less than 5 (HBD < 5) and HBA less than 10 (HBA < 10). Research in recent years has led to more precise guidelines, as described in detail by Krajl et al. [26].

The rules governing the passage of drug-like compounds across the BBB have been intensively studied. Van der Waterbeemd et al. indicated two rules for CNS-active compounds: MW ≤ 450 g mol^−1^, and TPSA ≤ 90 Å^2^ [20,27]. Ajay et al. [28] provided 5 rules: 200 g mol^−1^ ≤ MW ≤ 400 g mol^−1^, log P ≤ 5.2, HBA ≤ 4, HBD ≤ 3, and NRB ≤ 7. These rules allow the calculation and prediction of log BB values based on in silico physicochemical descriptors, both for CNS-active and CNS-inactive compounds [29].

The limits and rules presented above apply to molecules whose desired property is good penetration into the central nervous system. They serve as filters to separate molecules that affect the CNS from those with peripheral action, whose brain penetration, as an undesirable side effect, should be minimized as much as possible. Since the compounds we test are considered as potential drugs from both of these groups, it is important to predict their penetration through the blood-brain barrier, both effective and limited.

The in silico log BB values calculated on the basis of the structures of the tested compounds range from -1.315 to 0.772, and indicate that we are dealing with a fairly wide range of molecules with limited, moderate and high permeability through the BBB.

### 2.2. Establishment of Quantitative Structure (Retention)–Activity Relationships

The Quantitative Structure (Retention)–Activity Relationships (QS(R)ARs) method is based on linking the biological activity of compounds with chromatographic retention and/or with various physicochemical parameters characterizing these molecules. The basics of the method can be presented in the form of the following mathematical model (Equation (2)) [30,31,32]:

Biological activity = aA + bB + cC +⋯+ const = ƒ (lipophilic, electronic, steric properties)(2)

It is possible to predict the biological properties of compounds mainly from their chromatographic lipophilicities (retention coefficients). QRARs are useful when lipophilicity is the determining factor for the biological activity of the tested compound group [33], and there are good linear correlations between the retention and biological parameters [34,35]. Otherwise, additional parameters characterizing the compounds are introduced into the model as independent variables. The mathematical form of the model (QSARs) is obtained using multiple linear regression (MLR) [36]. The most frequently used descriptors describing the steric properties of a compound include: molar mass, molar volume, polarizability, parachor, and the number of rotatable bonds indicating the flexibility of the molecule [37,38]. The topological polar surface area, and the number of hydrogen bond donors and acceptors, are electronic descriptors [39,40,41,42]. The basic parameter characterizing the lipophilicity of the molecule is the log P_o/w_ (i.e., the logarithmic partition coefficient in the n-octanol/water system). The difficulties associated with measurements of the log P_o/w_ values mean that alternative lipophilic parameters, such as chromatographic retention parameters measured using the reversed phase liquid chromatography (RP LC) technique, are increasingly used, especially in screenings [43].

The aim of the QSARs method is to predict the pharmacokinetic properties of new, previously untested compounds. The derived models enable the prediction of such compounds’ activity with a high degree of probability, thereby facilitating the search for new molecules with desired properties. When establishing new relationships, it is important to use reliable and accurate data, choose descriptors judiciously, and validate the resulting model [36,44]. The descriptors (independent variables) used in the model must differentiate the tested compounds and cannot be interrelated [45]. The interrelationships of potential independent variables can be checked using cluster analysis or principal component analysis (PCA). The variance inflation factor (VIF) is also helpful. Calculating the coefficient of determination (R^2^) allows us to assess the fit of the obtained model to the output data. Adjusted R-squared (R^2^_adj_) and predicted R-squared (R^2^_pred_) are used to compare the fit of regression models that contain different numbers of independent variables, and to assess the reliability of the predictions made by the resulting model, respectively [46]. The derived QSARs models should be validated. The use of leave-out cross-validation allows the fit of the derived model to be checked by rejecting one or more variables from the data set and comparing the statistical parameters obtained for the relationships derived for both the full and the incomplete set. The applicability domain (AD) [47,48,49] indicates the area in which the model allows forecasting with good probability, and isolates outliers.

When deriving QSAR equations, it is necessary to limit the number of independent variables in the model, and remember that they cannot be intercorrelated. 

**Figure 1:**
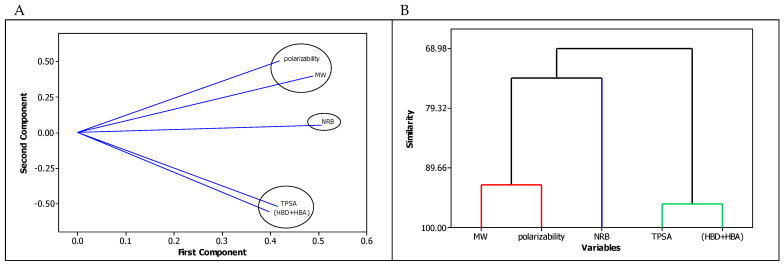
Similarities and dissimilarities between in silico descriptors of the tested compounds: (**A**) PCA—the loading plot, and (**B**) dendrogram.

In our investigations, the similarities (intercorrelations) between structural descriptors (considered as independent variables in QSARs models) were assessed based on PCA and cluster analysis of variables (Figure 1A,B). The loading plot (Figure 1A) indicates the similarity of the influence of individual variables on each component [50]. The sharper the “arrow” formed by the lines corresponding to individual variables, the greater their consistency in the assessment of the tested compounds. For polarizability and MW, the first component values are 0.42 and 0.49, respectively, and the second component values are 0.51 and 0.40, respectively. For TPSA and HBD + HBA, the first component values are 0.41 and 0.40, respectively, and the second component values are −0.52 and −0.56, respectively. Both the molar mass and polarizability of a molecule are related to its size, and the similarity between these descriptors is 92.57% (Figure 1B).

The polar surface area of a molecule, which is indicative of its ability to form hydrogen bonds, is typically calculated by summing the surface area contributions from oxygen and nitrogen atoms, as well as the hydrogen atoms attached to them. Consequently, it closely correlates with the number of hydrogen bond acceptors and hydrogen bond donors. As can be seen from Figure 1B, the similarity between TPSA and the numbers of hydrogen bond donors and acceptors is estimated at 95.48%. The increase of NRB results from the substituents introduced into the molecule and increases both its size (MW, polarizability) and the topological polar surface area due to the increased number of HBA (compounds from groups **IX** and **X**) or HBD (molecules from other groups). However, the similarity between NRB values and descriptors characterizing the size of the molecule and its polarity is much lower, and amounts to 74.05% and 68.98%, respectively (Figure 1B). In both graphs (Figure 1A,B) the same results are presented, and three clusters are indicated: the first for MW and polarizability, the second for TPSA and HBD + HBA, and the third for NRB.

Based on the similarity of the considered independent variables, possible QSAR models were derived, taking into account descriptors characterizing the compound’s lipophilicity (log k_w, IAM_), molecular size (MW or α), electronic properties (TPSA or HBD + HBA), and flexibility (NRB). These QSAR models were subsequently subjected to statistical evaluation (Table S2 in the Supplementary material). In our investigations, the following validation parameters were applied: the mean squared error (MSE), the coefficient of determination (R^2^), the determination coefficient adjusted (R^2^_adj_), and the determination coefficient predicted (R^2^_pred_). R^2^_adj_ is used to compare the goodness-of-fit for regression models that contain differing numbers of independent variables, while R^2^_pred_ determines how well a regression model makes predictions. The MSE is used to assess the predictive ability and accuracy of the model. The derived models were compared and assessed by leave-30%-out (i.e., 38 compounds) cross validation. Validation allows assessment of the quality of a QSAR model [51,52,53]. The resulting determination coefficient (Q^2^_cv_) and PRESS_cv_ were calculated, and are shown in Table 3. PRESS assesses a model’s predictive ability: the smaller the PRESS value, the better the model’s predictive ability [54]. A calculated global PRESS value lower than the sum of the squares of the response values of the total observations (SS) proves that the developed models predict better than chance [55]. Reasonable QSAR models should have Q^2^_cv_ values greater than 0.6, or a ratio of PRESS/SS smaller than 0.4 [54]. QSAR models are only valid in the domain in which they were validated [45,55], i.e., applicability domain (AD). AD is a space of information in which the model has been developed, and for which it is applicable to make predictions for new compounds. In the present work, we used the leverage approach (Williams plot) where the warning leverage, h*, was calculated according to Equation (3):

(3) $$h^{*}=\frac{3(p+1)}{n}$$

where n is the total number of samples in the dataset and p is the number of descriptors involved in the correlation [56].

Based on statistical evaluation, we have chosen the following model to predict the penetration of the investigated compounds across the BBB (Equation (4)):

log BB = −0.0004(0.097) + 0.0081(0.019)log k_w, IAM_ + 0.0044(0.000)MW − 0.2030(0.006) (HBD + HBA) (4)

The selected QSAR model has very good statistics and fulfills the principle of parsimony as it minimizes the number of descriptors (NRB values were omitted because they do not significantly affect the model; Table S2 in the Supplementary material). This model is based on three descriptors: the experimentally derived log k_w, IAM_ value; in silico calculated molecular weight; and the total number of hydrogen bond donors and hydrogen bond acceptors. The log k_w_ values determined on the IAM column ranged from −0.27 to 3.40; the molecular weights were within the range of 186.21–430.11; numbers of HBD and HBA ranged from 0 to 3, and from 4 to 10, respectively. It can be seen that in this model, high log BB values of the investigated molecules are favored by high log k_w, IAM_ and MW values, and a low content of HBD and HBA atoms. The inclusion of MW rather than α makes the model easier to apply. The polarizability of a molecule can also be calculated from the structure, but this requires expensive software which is not available in every laboratory.

**Figure 2:**
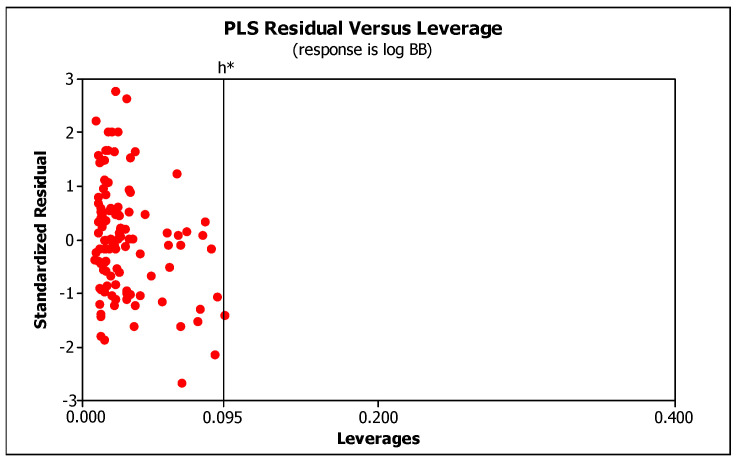
The Williams plot: applicability domain.

Table 3 contains the R^2^, R^2^_adj_, R^2^_pred_, PRESS, VIF, SS, MSE, F, p, and Q^2^ values calculated for the model. Very high R^2^ values (>>0.8) indicate that the model is very good. R^2^_pred_ and R^2^_adj_ values much higher than 0.8 also indicate the model’s high predictive ability. The ratio of PRESS/SS is much smaller than 0.4, and the variance inflation factor, VIF, does not exceed 5, which confirms moderate correlation of the descriptors. Statistics indicate that the model is very good and has a high predictive ability. Applicability domain (Figure 2) (limited by the values ± 3SD and the warning leverage, h* = 0.095) indicates that the model is valid within the domain it was developed. 

According to the derived model (Equation (4)), the penetration of the investigated compound through the blood-brain barrier depends on its lipophilicity (chromatographic lipophilicity log k_w, IAM_), molecular size, and its ability to form a hydrogen bond, i.e., the number of hydrogen bond donors and acceptors. As both the lipophilicity and the molar mass of a molecule increase, the presence of hydrogen bond donors and acceptors tends to weaken the compound’s penetration through the blood-brain barrier. These findings fully align with the data reported in the literature. Recognized criteria for the penetration of compounds into the brain refer primarily to the lipophilicity of the molecule. The positive effect of the lipophilicity of compounds on their penetration through the blood-brain barrier is quite well researched and defined [19,57,58]. Other criteria, like a limit of 8–10 hydrogen bonding groups per molecule, have also been proposed. Research by Abraham et al. indicates that the acidity and basicity of compounds (the number of hydrogen bond donors and acceptors) reduce the log BB values, i.e., make it more difficult to penetrate the blood-brain barrier [59,60]. The positive effect of molecular size on the log BB values could be explained by the partition mechanism of this process, and the positive effect of molecular size on non-specific van der Waals interactions [61].

The log BB models also contain descriptors relating to polarity or hydrogen bonding capacity (for example PSA), number of hydrogen-bond donors and acceptors, or hydrogen-bond acidity/basicity. In all cases, these descriptors correlate negatively with log BB. This implies that an increase in hydrogen-bonding strength or capacity typically results in reduced brain permeation, with highly polar or strongly hydrogen-bonding compounds having difficulty permeating membranes. Moreover, because water is known to be more acidic and more basic than the brain [62] due to its ability to form hydrogen bonds, both as an acceptor and a donor. It is in constant competition in these interactions with every other hydrogen bond donor and acceptor. 

**Figure 3:**
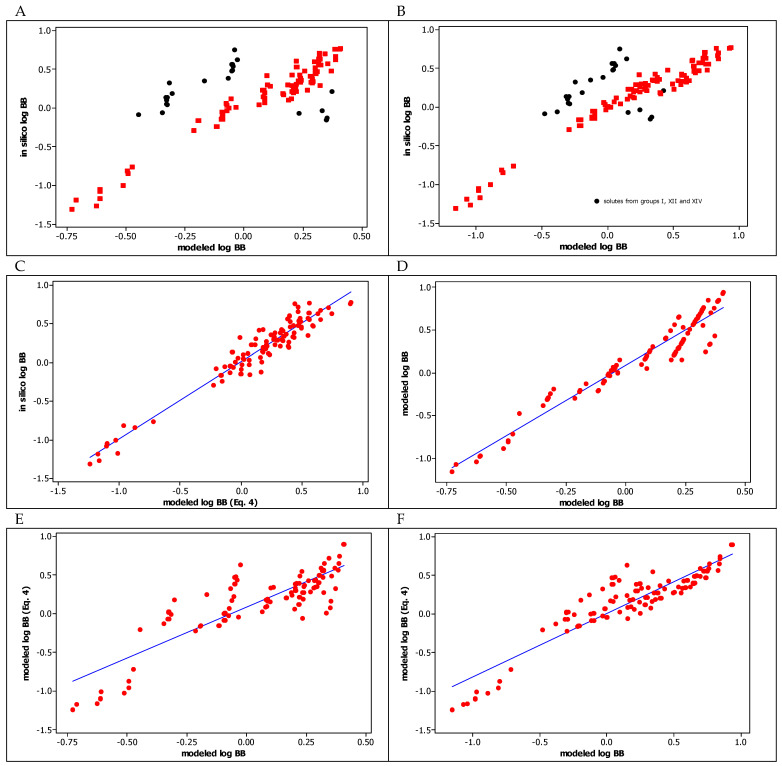
(**A**). Correlation between in silico log BB and modeled log BB [23] values. (**B**). Correlation between in silico log BB and modeled log BB [22] values. (**C**). Correlation between in silico log BB and modeled log BB (Equation (4)) values; R^2^ = 0.934. (**D**). Correlation between modeled log BB [22] and modeled log BB [23] values; R^2^ = 0.929. (**E**). Correlation between modeled log BB (Equation (4)) and modeled log BB [23] values; R^2^ = 0.742. (**F**). Correlation between modeled log BB (Equation (4)) and modeled log BB [22] values; R^2^ = 0.843.

In our previous studies, 65 [23] and 19 [22] heterocyclic structures from Table 1 were investigated using IAM column, and their BBB permeability was modeled by two equations (see Equation (13) in [23] and Equation (10) in [22]), which included chromatographic lipophilicity determined on the IAM column (log k_w, IAM_), and polarizability and TPSA as molecular size and polarity descriptors, respectively. All these models, previous and current, show a consistent effect of the same properties of the tested compounds on log BB values. Both the lipophilic nature of the compound (chromatographic lipophilicity log k_w, IAM_) and the size of the molecule (MW or polarizability) increase the log BB values, while its polarity (TPSA or the total number of hydrogen bond donors and acceptors) decreases the log BB values. The larger and the less polar the molecule is, the better it penetrates the BBB. In Figure 3, we present the correlations between the log BB parameters calculated on the basis of those models derived earlier and in the current work (Equation (4)) for all 126 tested compounds. As one can see, these models are consistent with each other, and the correlations between log BB values calculated according to the derived models (Figure 3D–F) are linear and good (R^2^ > 0.7) or very good (R^2^ > 0.9). Comparing to in silico log BB values, the current model seems to be the most universal as it encompasses all investigated heterocyclic molecules, including those from groups **I**, **XII** and **XIV** (Figure 3C), which exhibited some deviations in earlier models (Figure 3A,B). The greater general applicability of the new model (Equation (4)) results from the inclusion of compounds with a more diverse chemical nature, i.e., those with hydrogen bond donors in their molecules (classes **IX** and **X**). The results obtained indicate that the ability of the molecule to form hydrogen bonds (both as a donor and an acceptor) is an important factor influencing its penetration through the BBB. The sum of HBD and HBA in QSAR models is more useful for prediction of the log BB values than is the polarity (TPSA) of the molecule.

The QSAR model developed by us may have practical applicability in calculating the blood-brain barrier permeability of new heterocyclic drug-like small molecules. The only requirements for predicting the log BB of novel compounds using our model are to calculate their straightforward molecular descriptors, such as MW and the total number of HBD and HBA (which is even possible without the use of any computer software), and perform a simple chromatographic experiment to derive their log k_w, IAM_ values. Thus, the developed model may be conveniently applied in the design of new agents acting on the CNS. We recognize that our model has a high predictive power for small molecules that penetrate the blood-brain barrier by passive diffusion. However, it may have some limitations. It should not be used in the case of compounds known to be able to cross the blood-brain barrier via active transport or using efflux pumps. The log BB values of such molecules cannot be easily predicted from their physicochemical properties [63].

A number of our potential heterocyclic drugs possess a modeled log BB > 0.3 (derived from our QSAR model, Equation (4)), suggesting that they should readily cross the blood-brain barrier [64,65,66]. As possible drug candidates that are intended to act in the brain, they were designed to have different non-polar moieties, temporarily masked polar groups, and a minimum number of polar groups. It is worth noting that some heterocycles (i.e., structures **83**, **89**, **103**, **109**, **114** and **116**) with a modeled log BB in the range from 0.342 to 0.558, after preliminary tests in the central nervous system of mice, were found to have analgesic activity and low toxicity [21]. Therefore, further in vivo behavioral tests (on the CNS) of the remaining molecules—with log BB values suggesting that they are able to readily cross the BBB—are fully justified and planned for the future. The aim of such studies would be to compare the modeled data with those obtained experimentally. The ability to penetrate the BBB would be of importance in the case of their confirmed in vivo pharmacological efficiency in the CNS. However, heterocyclic molecules **53**, **56**–**60**, i.e., those containing hydrazide groups (which are hydrogen bond donors and electron acceptors) in their structures, showed a modeled log BB in the range from −1.014 to −1.241, suggesting that they would be poorly distributed to the brain [64,65,66]. This is as expected, because the ability to form hydrogen bonds is known to reduce penetration of the brain-blood barrier [59]. The most likely outcome is that hydrazides **56**–**60** with antioxidant and radical scavenging properties (as mentioned in the Supplementary material) should be deprived of CNS side effects due to their modeled log BB values of less than −1. However, it can be supposed that all our heterocyclic molecules with log BB below 0.3 would have limited access to the central nervous system. 

### 2.3. Assessing the Molecular Targets for Potential Pharmaceuticals Evaluated in This Paper (***1***–***126***)

Six molecular targets (such as G-protein coupled receptor (GPCR) and nuclear receptor ligands, enzyme, kinase and protease inhibitors, as well as ion channel modulators) for the potential drugs of small molecular weight reported in this study (**1**–**126**) were assessed by the use of Molinspiration Cheminformatics free web server (available online at www.molinspiration.com, accessed on 18 November 2023). For all the compounds, the bioactivity score with regard to these molecular targets was calculated in silico (Table S1 in the Supplementary material). Considering the predicted bioactivity, monosubstituted 5,6-dihydroimidazo[2,1-*c*][1,2,4]triazoles (**1**–**5**) are most likely to be active as GPCR-modulating ligands, enzyme inhibitors and ion channel modulators, whereas disubstituted 5,6-dihydroimidazo[2,1-*c*][1,2,4]triazoles (**6**–**14**) are most likely to be active as GPCR-modulating ligands, kinase inhibitors, enzyme inhibitors, and ion channel modulators. However, the latter can act on a variety of molecular targets. These in silico findings partially overlap with the literature data. The literature search disclosed that two imidazoloannelated 1,2,4-triazoles with antiproliferative effects in vitro were identified as tyrosine kinase inhibitors with high affinity to ephrin type-B receptor 3 (EPH-B3) and fibroblast growth factor receptor 1 (FGF-R1) [67,68,69]. In turn, taking into account the bioactivity scores for disubstituted 7,8-dihydroimidazo[2,1-*c*][1,2,4]triazin-4(6*H*)-ones (**15**–**126**), it can be seen that they are most likely to activate G-protein coupled receptors as GPCR-modulating ligands, and to inhibit enzymes and kinases. Disubstituted azoloanellated triazinones **61**–**70**, **81**–**92** and **122**–**126,** belonging to classes **XI**, **XIII** and **XVI**, respectively, are most likely to be active as GPCR ligands. This may be due to their structural similarity to the disclosed highly selective A_2B_ and A_2A_ adenosine receptor antagonists, which belong to the G-protein-coupled receptors [70]. The enzyme inhibition abilities predicted for the remaining azoloanellated triazinones may be as anticipated because of the similarity of these antimetabolite-type condensed aza-analogues of isocytosine to biogenic nucleobases. As false building blocks, they may participate in various metabolic pathways in which they lead to lethal synthesis by inhibiting enzymes [71]. However, the highest kinase inhibition abilities predicted for compounds **71**, **72**–**80**, **81**–**92** and **122**–**126**, from classes **XI**, **XII**, **XIII** and **XVI**, respectively, may result from the fact that some imidazoloannelated 1,2,4-triazines proved to be active in hematological malignancies as inhibitors of glycogen synthase kinase 3 [72].

## 3. Materials and Methods

### 3.1. Solvents and Reagents

Acetonitrile of HPLC grade (Merck, Lublin, Poland), anhydrous disodium phosphate and citric acid at the highest grade available (POCh, Lublin, Poland), and deionized water (Direct-Q3 UV system, Millipore, Warsaw, Poland), were used to prepare the buffered acetonitrile-water mixtures. 

### 3.2. Chromatography Equipment

High performance liquid chromatography (HPLC) measurements were performed on a Shimadzu Vp liquid chromatographic system (Shimadzu, Izabelin, Poland) [20]. This instrument was equipped with LC 10AT pump, SPD 10A ultraviolet-visible detector, SCL 10A system controller, CTO-10 AS chromatographic oven, and a Rheodyne injector valve with a 20 μL loop. As the stationary phase Regis IAM.PC.DD2 column, 100 × 4.6 mm i.d., 10 μm (Morton Grove, IL, USA) was applied. 

### 3.3. Chromatographic Conditions

Buffered acetonitrile mixtures were used as effluents. Acetonitrile concentration, expressed as a volume fraction, was varied within a range from 0.2 to 0.5, with a constant step of 0.1. The buffer was prepared from 0.01 mol L^−1^ solutions of Na_2_HPO_4_ and citric acid, and a pH value of 7.4 was fixed before mixing with an organic modifier. The flow rate was 1.3 mL min^−1^. Each compound sample was dissolved in acetonitrile to obtain a concentration of 0.005 mg mL^−1^, and each solute peak was detected under ultraviolet light at a wavelength of λ_max_ = 254 nm. Each measurement was performed at a constant temperature (25 °C) at least three times. The dead time values were measured from sodium chloride peaks. 

From among 126 heterocyclic compounds evaluated in this work (Table 1), 89 structures have been investigated previously on the IAM column: these are molecules from groups **III**, **IV**, **V**, **VI**, **VII**, **VIII**, **IX**, **XI**, **XIII**, and **XV** [21,22,23]. The remaining ones, i.e., 37 compounds from groups **I**, **II**, **X**, **XII** and **XIV**, were tested in this study for the first time using the biomimetic IAM chromatographic technique. To calculate the log k_w_ values (i.e., the logarithms of retention parameter in the buffer as the mobile phase), the retention parameters, logs k (k = (t_r_ − t_0_)/t_0_; where t_r_ is the solute retention time, and t_0_ is a dead time), that were measured for all tested compounds, were used according to Soczewiński-Wachtmeister’s equation (Equation (5)) [73]:

(5) $$\log\;k=\log\frac{t_{r}-t_{0}}{t_{0}}=\log\;k_{w}-s\varphi$$

where φ denotes the volume fraction of acetonitrile as organic modifier in the mobile phase, and k_w_ means the retention parameter corresponding to buffer as the mobile phase. The log k_w_ values were evaluated as linear regression coefficients (Table 2).

### 3.4. In Silico Studies

MW, TPSA, α, HBD, HBA, and NRB of the tested compounds, as well as parameters characterizing their distribution between the blood and the brain (log BB), were evaluated using ACD/Percepta software v. 2012 (Advanced Chemistry Development, Inc., Toronto, ON, Canada).

### 3.5. Statistical Analysis

Linear regression (LR), multiple linear regression (MLR), and cross validation were performed employing the statistical software package Minitab 16 (Minitab Inc., State College, PA, USA).

## 4. Conclusions

QSAR methodology was successful in modeling the BBB permeability of a large number of structurally diversified molecules. Biomimetic IAM chromatography was utilized to assess the chromatographic lipophilicity of 126 drug-like molecules being considered as potential pharmaceuticals. The QSARs model established by multiple linear regression showed a positive effect of the lipophilicity (log k_w, IAM_) and molecular weight of the compound, and a negative effect of the total number of hydrogen bond donors and acceptors, on the log BB values. It confirmed that for the drug-like compounds meeting not only Ro5, but also more precise requirements, an increase in lipophilicity and molecular size favors their penetration through the blood-brain barrier, while acidity and/or basicity hinder it. The model derived from both in vitro (chromatographic indices) and in silico (molecular descriptors) data is not excessively complicated, can be applied in the design of new agents active in the CNS, and serves as a guide for further research. It acts as a filter to select the most promising compounds and predict the potential side effects of their action. This model may be helpful in the research of pharmacologists interested in calculating the passive blood-brain barrier permeability of new drug-like compounds because it allows the estimation of log BB using a simple chromatographic experiment and easily-calculated physicochemical descriptors, which can be obtained without the use of complicated and expensive computer programs. The research findings underscore the significance of biomimetic chromatographic techniques which, when combined with QSAR modeling, offer an opportunity to reduce or eliminate costly and unethical animal testing in screening studies for bioactive molecules. The results of these studies will be very important in further in vivo investigations during drug development. The possible molecular targets for the potential pharmaceuticals investigated in this study were assessed in silico.

## Figures and Tables

**Table 1:** Heterocyclic potential drugs (**1**–**126**) from classes **I**–**XVI**, serving as a set for testing blood-brain barrier permeability in the present study.

Class	General Structure	No.	R_1_	R_2_
**I**	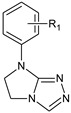	**1** **2** **3** **4** **5**	R_1_ = H R_1_ = 4-CH_3_ R_1_ = 4-OCH_3_ R_1_ = 3-Cl R_1_ = 3,4-Cl_2_	
**II**	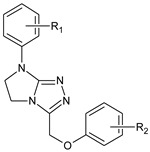	**6** **7** **8** **9** **10** **11** **12** **13** **14**	R_1_ = H R_1_ = 4-OCH_3_ R_1_ = H R_1_ = 4-CH_3_ R_1_ = 4-Cl R_1_ = 4-Cl R_1_ = 4-Cl R_1_ = 3,4-Cl_2_ R_1_ = 4-Cl	R_2_ = H R_2_ = 4-Cl R_2_ = 2-CH_3_; 4-Cl R_2_ = 2-CH_3_; 4-Cl R_2_ = 4-Cl R_2_ = 2-CH_3_; 4-Cl R_2_ = 2,4-Cl_2_ R_2_ = 2,4-Cl_2_ R_2_ = 2,4,5-Cl_3_
**III**	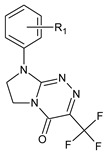	**15** **16** **17** **18** **19** **20** **21** **22**	R_1_ = H R_1_ = 2-CH_3_ R_1_ = 4-CH_3_ R_1_ = 2-OCH_3_ R_1_ = 2-Cl R_1_ = 3-Cl R_1_ = 4-Cl R_1_ = 3,4-Cl_2_	
**IV**	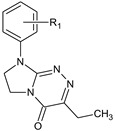	**23** **24** **25** **26** **27** **28**	R_1_ = H R_1_ = 4-CH_3_ R_1_ = 2-Cl R_1_ = 3-Cl R_1_ = 4-Cl R_1_ = 3,4-Cl_2_	
**V**	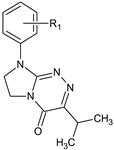	**29** **30** **31** **32** **33** **34**	R_1_ = H R_1_ = 4-CH_3_ R_1_ = 2-Cl R_1_ = 3-Cl R_1_ = 4-Cl R_1_ = 3,4-Cl_2_	
**VI**	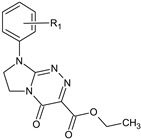	**35** **36** **37** **38** **39**	R_1_ = H R_1_ = 4-CH_3_ R_1_ = 3-Cl R_1_ = 4-Cl R_1_ = 3,4-Cl_2_	
**VII**	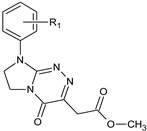	**40** **41** **42** **43** **44**	R_1_ = H R_1_ = 4-CH_3_ R_1_ = 4-OCH_3_ R_1_ = 4-OC_2_H_5_ R_1_ = 4-Cl	
**VIII**	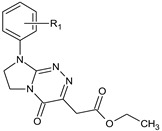	**45** **46** **47** **48** **49** **50**	R_1_ = H R_1_ = 4-CH_3_ R_1_ = 2-OCH_3_ R_1_ = 3-Cl R_1_ = 4-Cl R_1_ = 3,4-Cl_2_	
**IX**	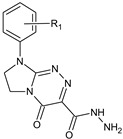	**51** **52** **53** **54** **55**	R_1_ = H R_1_ = 4-CH_3_ R_1_ = 4-OCH_3_ R_1_ = 3-Cl R_1_ = 3,4-Cl_2_	
**X**	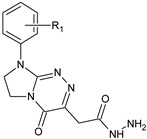	**56** **57** **58** **59** **60**	R_1_ = H R_1_ = 4-CH_3_ R_1_ = 4-OCH_3_ R_1_ = 4-OC_2_H_5_ R_1_ = 4-Cl	
**XI**	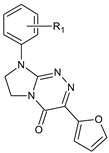	**61** **62** **63** **64** **65** **66** **67** **68** **69** **70** **71**	R_1_ = H R_1_ = 2-CH_3_ R_1_ = 4-CH_3_ R_1_ = 2,3-(CH_3_)_2_ R_1_ = 2-OCH_3_ R_1_ = 4-OCH_3_ R_1_ = 2-Cl R_1_ = 3-Cl R_1_ = 4-Cl R_1_ = 3,4-Cl_2_ R_1_ = 2,6-Cl_2_	
**XII**	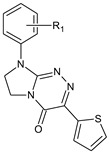	**72** **73** **74** **75** **76** **77** **78** **79** **80**	R_1_ = H R_1_ = 2-CH_3_ R_1_ = 4-CH_3_ R_1_ = 2,3-(CH_3_)_2_ R_1_ = 2-OCH_3_ R_1_ = 2-Cl R_1_ = 3-Cl R_1_ = 4-Cl R_1_ = 3,4-Cl_2_	
**XIII**	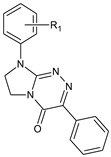	**81** **82** **83** **84** **85** **86** **87** **88** **89** **90** **91** **92**	R_1_ = H R_1_ = 2-CH_3_ R_1_ = 3-CH_3_ R_1_ = 4-CH_3_ R_1_ = 2-OCH_3_ R_1_ = 4-OCH_3_ R_1_ = 4-OC_2_H_5_ R_1_ = 2,3-(CH_3_)_2_ R_1_ = 2-Cl R_1_ = 3-Cl R_1_ = 4-Cl R_1_ = 3,4-Cl_2_	
**XIV**	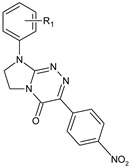	**93** **94** **95** **96** **97** **98** **99** **100** **101**	R_1_ = H R_1_ = 2-CH_3_ R_1_ = 4-CH_3_ R_1_ = 2-OCH_3_ R_1_ = 2,3-(CH_3_)_2_ R_1_ = 2-Cl R_1_ = 3-Cl R_1_ = 4-Cl R_1_ = 3,4-Cl_2_	
**XV**	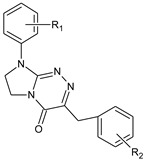	**102** **103** **104** **105** **106** **107** **108** **109** **110** **111** **112** **113** **114** **115** **116** **117** **118** **119** **120** **121**	R_1_ = H R_1_ = H R_1_ = H R_1_ = H R_1_ = 4-CH_3_ R_1_ = 4-CH_3_ R_1_ = 4-CH_3_ R_1_ = 4-CH_3_ R_1_ = 4-CH_3_ R_1_ = 4-CH_3_ R_1_ = 4-OC_2_H_5_ R_1_ = 4-OC_2_H_5_ R_1_ = 4-OC_2_H_5_ R_1_ = 4-OC_2_H_5_ R_1_ = 4-OC_2_H_5_ R_1_ = 2-CH_3_ R_1_ = 4-Cl R_1_ = 4-Cl R_1_ = 4-Cl R_1_ = 4-Cl	R_2_ = H R_2_ = 2-Cl R_2_ = 3-Cl R_2_ = 4-Cl R_2_ = H R_2_ = 4-CH_3_ R_2_ = 3-CH_3_ R_2_ = 2-Cl R_2_ = 3-Cl R_2_ = 4-Cl R_2_ = H R_2_ = 4-CH_3_ R_2_ = 2-Cl R_2_ = 3-Cl R_2_ = 4-Cl R_2_ = 2-Cl R_2_ = H R_2_ = 2-Cl R_2_ = 3-Cl R_2_ = 4-Cl
**XVI**	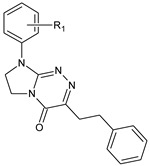	**122** **123** **124** **125** **126**	R_1_ = H R_1_ = 4-CH_3_ R_1_ = 2-Cl R_1_ = 4-Cl R_1_ = 3,4-Cl_2_	

**Table 2:** Molecular descriptors (MW, NRB, HBD, HBA, TPSA, α), log BB, and log k_w, IAM_ parameters of tested compounds.

Compound	MW [g mol^−1^]	NRB	HBD	HBA	TPSA [Å^2^]	α [Å^3^]	log BB	log k_w, IAM_
**1**	186.21	1	0	4	33.95	21.80	−0.033	0.10
**2**	200.24	1	0	4	33.95	23.56	−0.159	0.41
**3**	216.24	2	0	5	43.18	24.11	−0.068	0.10
**4**	220.66	1	0	4	33.95	23.63	−0.128	0.64
**5**	255.10	1	0	4	33.95	25.45	0.209	1.18
**6**	292.34	4	0	5	43.18	34.13	0.292	0.11
**7**	356.81	5	0	6	52.41	38.26	0.340	0.06
**8**	340.81	4	0	5	43.18	37.71	0.571	0.09
**9**	354.83	4	0	5	43.18	39.47	0.763	1.95
**10**	361.22	4	0	5	43.18	37.78	0.476	1.86
**11**	375.25	4	0	5	43.18	39.54	0.668	2.14
**12**	395.67	4	0	5	43.18	39.61	0.626	2.25
**13**	430.11	4	0	5	43.18	41.43	0.772	2.91
**14**	430.11	4	0	5	43.18	41.43	0.758	2.55
**15** ^a^	282.22	2	0	5	48.27	25.85	0.102	0.94
**16** ^a^	296.25	2	0	5	48.27	27.61	0.290	0.76
**17** ^a^	296.25	2	0	5	48.27	27.61	0.290	1.25
**18** ^a^	312.25	3	0	6	57.50	28.16	0.063	0.67
**19** ^a^	316.67	2	0	5	48.27	27.68	0.211	0.88
**20** ^a^	316.67	2	0	5	48.27	27.68	0.264	1.66
**21** ^a^	316.67	2	0	5	48.27	27.68	0.194	1.58
**22** ^a^	351.11	2	0	5	48.27	29.50	0.345	2.29
**23** ^b^	242.28	2	0	5	48.27	27.55	0.117	0.55
**24** ^b^	256.30	2	0	5	48.27	29.30	0.305	1.00
**25** ^b^	276.72	2	0	5	48.27	29.37	0.226	0.80
**26** ^b^	276.72	2	0	5	48.27	29.37	0.270	0.84
**27** ^b^	276.72	2	0	5	48.27	29.37	0.209	1.24
**28** ^b^	311.17	2	0	5	48.27	31.20	0.360	1.85
**29** ^a^	256.30	2	0	5	48.27	29.30	0.230	0.76
**30** ^a^	270.33	2	0	5	48.27	31.06	0.423	1.05
**31** ^a^	290.75	2	0	5	48.27	31.13	0.339	0.65
**32** ^a^	290.75	2	0	5	48.27	31.13	0.384	1.49
**33** ^a^	290.75	2	0	5	48.27	31.13	0.328	1.39
**34** ^a^	325.19	2	0	5	48.27	32.95	0.473	1.67
**35** ^a^	286.29	4	0	7	74.57	30.27	−0.243	0.48
**36** ^a^	300.31	4	0	7	74.57	32.02	−0.051	1.93
**37** ^a^	320.73	4	0	7	74.57	32.09	−0.090	1.73
**38** ^a^	320.73	4	0	7	74.57	32.09	−0.151	1.12
**39** ^a^	355.18	4	0	7	74.57	33.91	0.000	3.36
**40** ^b^	286.29	4	0	7	74.57	30.27	−0.243	0.81
**41** ^b^	300.31	4	0	7	74.57	32.02	−0.051	1.33
**42** ^b^	316.31	5	0	8	83.80	32.57	−0.293	0.81
**43** ^b^	330.34	6	0	8	83.80	34.40	−0.167	1.42
**44** ^b^	320.73	4	0	7	74.57	32.09	−0.151	1.82
**45** ^b^	300.31	5	0	7	74.57	32.09	−0.132	1.21
**46** ^b^	314.34	5	0	7	74.57	33.85	0.055	1.70
**47** ^b^	330.34	6	0	8	83.80	34.40	−0.164	0.91
**48** ^b^	334.76	5	0	7	74.57	33.92	0.029	2.05
**49** ^b^	334.76	5	0	7	74.57	33.92	−0.033	2.11
**50** ^b^	369.20	5	0	7	74.57	35.74	0.117	2.42
**51** ^c^	274.28	2	3	8	103.39	28.32	−1.002	−0.14
**52** ^c^	288.31	2	3	8	103.39	30.07	−0.815	0.16
**53** ^c^	304.30	3	3	9	112.62	30.62	−1.050	−0.10
**54** ^c^	308.72	2	3	8	103.39	30.14	−0.845	0.41
**55** ^c^	343.17	2	3	8	103.39	31.96	−0.764	0.69
**56**	288.26	4	3	9	112.62	28.65	−1.272	0.40
**57**	302.29	4	3	9	112.62	30.41	−1.084	0.13
**58**	318.29	5	3	10	121.85	30.96	−1.315	−0.27
**59**	332.31	6	3	10	121.85	32.79	−1.189	0.06
**60**	322.71	4	3	9	112.62	30.48	−1.173	0.32
**61** ^b^	280.28	2	0	6	61.41	30.82	0.038	1.29
**62** ^b^	294.31	2	0	6	61.41	32.57	0.225	0.96
**63** ^b^	294.31	2	0	6	61.41	32.57	0.225	1.69
**64** ^b^	308.33	2	0	6	61.41	34.33	0.417	1.42
**65** ^b^	310.31	3	0	7	70.64	33.12	0.006	1.11
**66** ^b^	310.31	3	0	7	70.64	33.12	0.003	1.19
**67** ^b^	314.73	2	0	6	61.41	32.64	0.141	1.27
**68** ^b^	314.73	2	0	6	61.41	32.64	0.194	2.08
**69** ^b^	314.73	2	0	6	61.41	32.64	0.130	2.02
**70** ^b^	349.17	2	0	6	61.41	34.47	0.280	3.23
**71** ^b^	349.17	2	0	6	61.41	34.47	0.297	1.70
**72**	300.38	2	0	5	73.57	33.36	0.383	1.64
**73**	314.41	2	0	5	73.57	35.12	0.562	1.54
**74**	314.41	2	0	5	73.57	35.12	0.562	1.94
**75**	328.43	2	0	5	73.57	36.87	0.754	1.17
**76**	330.41	3	0	6	82.80	35.67	0.351	1.50
**77**	334.82	2	0	5	73.57	35.19	0.483	1.75
**78**	334.82	2	0	5	73.57	35.19	0.536	2.26
**79**	334.82	2	0	5	73.57	35.19	0.475	1.53
**80**	369.27	2	0	5	73.57	37.01	0.625	2.68
**81** ^b^	290.32	2	0	5	48.27	33.92	0.227	1.91
**82** ^b^	304.35	2	0	5	48.27	35.68	0.407	1.53
**83** ^b^	304.35	2	0	5	48.27	35.68	0.407	2.26
**84** ^b^	304.35	2	0	5	48.27	35.68	0.407	2.22
**85** ^b^	320.35	3	0	6	57.50	36.23	0.188	1.47
**86** ^b^	320.35	3	0	6	57.50	36.23	0.172	1.71
**87** ^b^	334.37	4	0	6	57.50	38.05	0.298	2.14
**88** ^b^	318.37	2	0	5	48.27	37.43	0.594	1.85
**89** ^b^	324.76	2	0	5	48.27	35.75	0.331	1.73
**90** ^b^	324.76	2	0	5	48.27	35.75	0.376	2.64
**91** ^b^	324.76	2	0	5	48.27	35.75	0.319	2.57
**92** ^b^	359.21	2	0	5	48.27	37.57	0.465	3.26
**93**	335.32	3	0	8	97.10	36.17	−0.059	1.92
**94**	349.34	3	0	8	97.10	37.92	0.133	2.52
**95**	349.34	3	0	8	97.10	37.92	0.133	1.83
**96**	365.34	4	0	9	106.33	38.47	−0.086	1.78
**97**	363.37	3	0	8	97.10	39.67	0.320	1.78
**98**	369.76	3	0	8	97.10	37.99	0.049	2.16
**99**	369.76	3	0	8	97.10	37.99	0.102	2.02
**100**	369.76	3	0	8	97.10	37.99	0.038	2.52
**101**	404.21	3	0	8	97.10	39.81	0.183	3.22
**102** ^b^	304.35	3	0	5	48.27	35.75	0.341	3.26
**103** ^b^	338.79	3	0	5	48.27	37.57	0.459	2.34
**104** ^b^	338.79	3	0	5	48.27	37.57	0.459	2.48
**105** ^b^	338.79	3	0	5	48.27	37.57	0.459	2.39
**106** ^b^	318.37	3	0	5	48.27	37.50	0.524	2.15
**107** ^b^	332.40	3	0	5	48.27	39.26	0.712	2.51
**108** ^b^	332.40	3	0	5	48.27	39.26	0.712	2.59
**109** ^b^	352.82	3	0	5	48.27	39.33	0.635	2.65
**110** ^b^	352.82	3	0	5	48.27	39.33	0.635	2.78
**111** ^b^	352.82	3	0	5	48.27	39.33	0.635	2.81
**112** ^b^	348.40	5	0	6	57.50	39.88	0.424	2.11
**113** ^b^	362.43	5	0	6	57.50	41.63	0.602	2.43
**114** ^b^	382.84	5	0	6	57.50	41.70	0.526	2.51
**115** ^b^	382.84	5	0	6	57.50	41.70	0.526	2.73
**116** ^b^	382.84	5	0	6	57.50	41.70	0.526	2.72
**117** ^b^	352.82	3	0	5	48.27	39.33	0.635	2.02
**118** ^b^	338.79	3	0	5	48.27	37.57	0.440	2.52
**119** ^b^	373.24	3	0	5	48.27	39.40	0.551	3.22
**120** ^b^	373.24	3	0	5	48.27	39.40	0.551	3.15
**121** ^b^	373.24	3	0	5	48.27	39.40	0.551	3.17
**122** ^b^	318.37	4	0	5	48.27	37.58	0.459	2.07
**123** ^b^	332.40	4	0	5	48.27	39.33	0.651	2.41
**124** ^b^	352.82	4	0	5	48.27	39.40	0.567	1.81
**125** ^b^	352.82	4	0	5	48.27	39.40	0.551	2.68
**126** ^b^	387.26	4	0	5	48.27	41.22	0.701	3.40

^a^—MW, TPSA, α, log BB and log k_w, IAM_ from Ref. [22]; ^b^—MW, TPSA, α and log k_w, IAM_ from Ref. [23]; ^c^—log k_w, IAM_ from Ref. [21].

**Table 3:** Statistics of the derived QSAR model (Equation (4)).

R^2^	R^2^_adj_	R^2^_pred_	PRESS	VIF	SS	MSE	F	p	Q^2^_cv_	PRESS_cv_
0.9342	0.9326	0.9296	1.76752	<2.8	25.1199	0.01355	578	0.0000	0.9342	1.69843

## Data Availability

Data are contained within the article and supplementary materials.

## Supplementary Materials

The following supporting information can be downloaded at: www.mdpi.com/xxx/s1, 1. The pharmacological and analytical significance of the investigated compounds (**1**–**126**). Table S1. The bioactivity score of the investigated compounds (**1**–**126**) for various molecular targets. 2. Establishment of the QSAR model. Table S2. Statistics of the derived QSAR models.
