# Supplementary material for: Modeling the Blood-Brain Barrier Permeability of Potential Heterocyclic Drugs via Biomimetic IAM Chromatography Technique Combined with QSAR Methodology
Source: Molecules. 2024 Jan 5;29(2):0. doi: 10.3390/molecules29020287 (PMC11154582; doi:10.3390/molecules29020287)
Supplement: Supplementary file 1 [file molecules-29-00287-s001.zip › molecules-2769178-supplementary.pdf]

molecules

Supplementary material

# Modeling the blood-brain barrier permeability of potential heterocyclic drugs *via* biomimetic IAM chromatography technique combined with QSAR methodology

**Małgorzata Janicka [1], Małgorzata Sztanke [2] and Krzysztof Sztanke [3]***

[1] Department of Physical Chemistry, Faculty of Chemistry, Institute of Chemical Science, Maria Curie-Skłodowska University, Lublin, Poland, malgorzata.janicka@mail.umcs.pl
[2] Department of Medical Chemistry, Medical University of Lublin, 4A Chodźki Street, 20-093 Lublin, Poland, malgorzata.sztanke@umlub.pl
[3] Laboratory of Bioorganic Compounds Synthesis and Analysis, Medical University of Lublin, 4A Chodźki Street, 20-093 Lublin, Poland
* Correspondence: krzysztof.sztanke@umlub.pl

## 1. *The pharmacological and analytical significance of the investigated compounds (**1-126**)*

A large number of drug candidates (**1-126**; classes **I-XVI**, Table 1) [1-33], were used as an important set in our current studies. All these compounds have small molecular weights. The privileged scaffold of 5,6-dihydroimidazo[2,1-*c*][1,2,4]triazole with a common N4-C7a bond is present in monosubstituted heterocyclic structures (class **I**), and disubstituted heterocycles (class **II**). In turn, the privileged template of 7,8-dihydroimidazo[2,1-*c*][1,2,4]triazin-4(6*H*)-one with a common N5-C8a bond is present in all the compounds belonging to the remaining classes (**III-XVI**). These two privileged scaffolds are already known in medicinal chemistry because of their important pharmacological activities [1,2].

The structures of all the molecules had been established in our earlier studies with the use of spectroscopic techniques [3-16], before the first-line evidence data indicated that they may have potential as future drugs, due to their significant antitumor (compounds **8-12**, **15-34**, **37**, **39**, **45-51**, **54**, **61-80**, **86**, **90-101**, **109**, **114**, **116**, **122-126**) [3-7,9-15,17-21], antimetastatic (compounds **83**, **86**, **89-91**) [14], antiviral (compound **28**) [19], antibacterial (compounds **9**, **13**) [3], analgesic (compounds **41**, **42**, **51**, **53**, **83**, **89**, **91**, **92**, **103**, **109**, **112**, **114**) [8,16], antihemolytic (compounds **15-34**, **46-48**, **83**, **89**, **92**) [4,19,26,30] and antiradical (compounds **53**, **55**, **58**) [21,22] activities. Some of these potential drugs have been reported to possess the elucidated mechanism of action: antiproliferative in human cancer cells – by inducing DNA damage (compounds **8**, **54**) or by promoting apoptotic caspases (compounds **16**, **18**, **19**, **31**, **33**, **34**), apoptosis (compounds **45**, **48**, **50**, **54**, **80**) and necrosis (compounds **45**, **48**, **50**, **80**); and antiradical – as the scavenging ability of DPPH radical (azoloannelated 1,2,4-triazinylcarbohydrazides **51-55** and fused 1,2,4-triazinylacetohydrazides **56-60**) [3,9,10,13,18-21]. The analgesically active and low in toxicity carbohydrazide **53**, has shown the antagonistic action towards hepatic adenosine $A_{2A}$ receptors (manifested by the ability to antagonize the stimulatory effect of a selective $A_{2A}$ receptor agonist), and has been able to scavenge and thus neutralize the superoxide anion radical. These beneficial features warrant its possible use in the treatment and prevention of liver cirrhosis [22]. Results of *in vitro*/*in vivo*/*ex vivo* studies have demonstrated that many of compounds have low or no toxicity to normal cell lines/zebrafish embryos and larvae/mice/erythrocytes [3,4,6-10,12-16,18-20]. Compounds **15**, **16**, **18-20** and **29-33** have revealed lower toxicity for zebrafish embryos and larvae than anticancer agent pemetrexed [18,20], while molecules **23-25** than antiviral agent

acyclovir [19]. In addition, compounds **15-25** and **29-31** have been shown to possess the stronger protective effect on oxidatively-stressed erythrocytes than ascorbic acid or trolox [4,19]. The vast majority of molecules from classes **III-V**, **XII**, **XIV** and **XVI** have revealed higher antitumor effects than pemetrexed [4,6,13,15]. The molecule **9** has been shown to possess the antibacterial activity (against *Staphylococcus aureus* ATCC 25923) comparable to that of ampicillin. In turn, the compound **13**, revealing the antibacterial potency (against *Pseudomonas aeruginosa* ATCC 25923) superior to that of chloramphenicol and ampicillin, has demonstrated the selective activity on Gram-negative strains of bacteria [3]. Thus, this molecule may be helpful in fighting against some bacterial strains that are resistant to known antibiotics. In addition, compounds that have been extensively thermally tested so far – from classes **I-III**, **V**, **VIII**, **IX**, **XI-XIV** – have revealed high thermal stability [23-31], and should therefore be suitable candidates for pharmaceutical use. In turn, molecules from the remaining classes are now submitted to such thermal investigations. For some potential pharmaceuticals (compounds **48**, **53**, **91** and **97**) selected from particular classes (**VIII**, **IX**, **XIII** and **XIV**), we have already developed the first analytical procedures (enabling their detection and quantitative determination in solutions and biological samples), which would have a chance for use in clinical analytics [32-35]. Currently, the development of such analytical methods for determining the most promising compounds from another classes is being continued.

**Table S1.** The bioactivity score of the investigated compounds (**1-126**) for various molecular targets.

| | Bioactivity Score | | | | | |
|---|---|---|---|---|---|---|
| **Compound** | **GPCR Ligand** | **Enzyme Inhibitor** | **Kinase Inhibitor** | **Ion Channel Modulator** | **Nuclear Receptor Ligand** | **Protease Inhibitor** |
| **1** | -0.53 | -0.74 | -1.07 | -0.78 | -1.50 | -1.42 |
| **2** | -0.50 | -0.74 | -1.01 | -0.82 | -1.37 | -1.36 |
| **3** | -0.42 | -0.64 | -0.86 | -0.75 | -1.19 | -1.22 |
| **4** | -0.45 | -0.73 | -0.94 | -0.71 | -1.38 | -1.39 |
| **5** | -0.34 | -0.64 | -0.81 | -0.63 | -1.20 | -1.24 |
| **6** | -0.14 | -0.53 | -0.48 | -0.57 | -0.68 | -0.68 |
| **7** | -0.14 | -0.54 | -0.44 | -0.56 | -0.63 | -0.63 |
| **8** | -0.15 | -0.59 | -0.49 | -0.60 | -0.60 | -0.70 |
| **9** | -0.17 | -0.59 | -0.50 | -0.61 | -0.60 | -0.68 |
| **10** | -0.12 | -0.53 | -0.44 | -0.53 | -0.62 | -0.62 |
| **11** | -0.15 | -0.56 | -0.47 | -0.58 | -0.57 | -0.66 |
| **12** | -0.13 | -0.53 | -0.38 | -0.56 | -0.60 | -0.66 |
| **13** | -0.13 | -0.54 | -0.37 | -0.54 | -0.59 | -0.67 |
| **14** | -0.11 | -0.51 | -0.38 | -0.55 | -0.56 | -0.63 |
| **15** | -0.30 | -0.36 | -0.50 | -0.69 | -0.77 | -0.85 |
| **16** | -0.33 | -0.38 | -0.44 | -0.73 | -0.75 | -0.87 |
| **17** | -0.31 | -0.42 | -0.50 | -0.74 | -0.73 | -0.84 |
| **18** | -0.30 | -0.36 | -0.41 | -0.81 | -0.70 | -0.86 |
| **19** | -0.19 | -0.41 | -0.32 | -0.66 | -0.77 | -0.84 |
| **20** | -0.27 | -0.41 | -0.46 | -0.67 | -0.73 | -0.86 |
| **21** | -0.26 | -0.39 | -0.46 | -0.66 | -0.72 | -0.83 |
| **22** | -0.22 | -0.39 | -0.41 | -0.63 | -0.66 | -0.79 |
| **23** | -0.54 | -0.46 | -0.79 | -0.92 | -1.18 | -1.21 |
| **24** | -0.52 | -0.49 | -0.76 | -0.96 | -1.09 | -1.17 |

| | | | | | | |
|---|---|---|---|---|---|---|
| **25** | -0.39 | -0.48 | -0.56 | -0.88 | -1.14 | -1.17 |
| **26** | -0.48 | -0.49 | -0.71 | -0.88 | -1.10 | -1.19 |
| **27** | -0.47 | -0.46 | -0.72 | -0.88 | -1.08 | -1.15 |
| **28** | -0.40 | -0.45 | -0.63 | -0.83 | -0.98 | -1.09 |
| **29** [a] | -0.41 | -0.37 | -0.61 | -0.77 | -0.95 | -1.00 |
| **30** [a] | -0.40 | -0.41 | -0.59 | -0.82 | -0.89 | -0.98 |
| **31** [a] | -0.28 | -0.41 | -0.40 | -0.74 | -0.93 | -0.98 |
| **32** [a] | -0.36 | -0.41 | -0.55 | -0.74 | -0.89 | -1.00 |
| **33** [a] | -0.35 | -0.39 | -0.56 | -0.74 | -0.88 | -0.96 |
| **34** [a] | -0.30 | -0.39 | -0.48 | -0.70 | -0.80 | -0.91 |
| **35** | -0.48 | -0.52 | -0.68 | -0.80 | -0.94 | -0.99 |
| **36** | -0.48 | -0.56 | -0.68 | -0.84 | -0.90 | -0.98 |
| **37** | -0.44 | -0.56 | -0.64 | -0.78 | -0.91 | -1.00 |
| **38** | -0.43 | -0.54 | -0.64 | -0.77 | -0.90 | -0.96 |
| **39** | -0.40 | -0.54 | -0.59 | -0.73 | -0.84 | -0.93 |
| **40** | -0.45 | -0.40 | -0.82 | -0.85 | -0.90 | -0.91 |
| **41** | -0.45 | -0.44 | -0.81 | -0.89 | -0.86 | -0.90 |
| **42** | -0.41 | -0.40 | -0.75 | -0.84 | -0.78 | -0.82 |
| **43** | -0.44 | -0.44 | -0.78 | -0.84 | -0.73 | -0.81 |
| **44** | -0.41 | -0.42 | -0.78 | -0.82 | -0.85 | -0.88 |
| **45** | -0.46 | -0.42 | -0.81 | -0.83 | -0.84 | -0.86 |
| **46** | -0.47 | -0.47 | -0.82 | -0.87 | -0.82 | -0.86 |
| **47** | -0.47 | -0.42 | -0.74 | -0.93 | -0.81 | -0.89 |
| **48** | -0.44 | -0.46 | -0.78 | -0.81 | -0.82 | -0.88 |
| **49** | -0.43 | -0.44 | -0.79 | -0.80 | -0.81 | -0.85 |
| **50** | -0.41 | -0.44 | -0.74 | -0.77 | -0.77 | -0.83 |
| **51** | -0.60 | -0.54 | -0.68 | -1.45 | -1.55 | -1.02 |
| **52** | -0.59 | -0.59 | -0.68 | -1.47 | -1.47 | -1.00 |
| **53** | -0.54 | -0.55 | -0.61 | -1.41 | -1.35 | -0.92 |
| **54** | -0.55 | -0.58 | -0.63 | -1.39 | -1.47 | -1.02 |
| **55** | -0.49 | -0.55 | -0.57 | -1.32 | -1.37 | -0.94 |
| **56** | -0.60 | -0.44 | -0.68 | -1.28 | -1.57 | -1.00 |
| **57** | -0.60 | -0.49 | -0.68 | -1.31 | -1.50 | -0.98 |
| **58** | -0.56 | -0.46 | -0.62 | -1.26 | -1.40 | -0.92 |
| **59** | -0.57 | -0.48 | -0.65 | -1.22 | -1.32 | -0.89 |
| **60** | -0.55 | -0.46 | -0.64 | -1.23 | -1.49 | -0.97 |
| **61** | -0.16 | -0.42 | -0.49 | -0.86 | -0.97 | -1.02 |
| **62** | -0.20 | -0.44 | -0.44 | -0.90 | -0.94 | -1.03 |
| **63** | -0.18 | -0.47 | -0.50 | -0.91 | -0.93 | -1.01 |
| **64** | -0.19 | -0.46 | -0.46 | -0.91 | -0.88 | -0.98 |
| **65** | -0.19 | -0.42 | -0.42 | -0.97 | -0.90 | -1.02 |
| **66** | -0.16 | -0.44 | -0.46 | -0.88 | -0.85 | -0.94 |
| **67** | -0.07 | -0.46 | -0.33 | -0.83 | -0.97 | -1.00 |

| | | | | | | |
|---|---|---|---|---|---|---|
| **68** | -0.15 | -0.47 | -0.46 | -0.84 | -0.93 | -1.02 |
| **69** | -0.14 | -0.47 | -0.47 | -0.83 | -0.92 | -0.99 |
| **70** | -0.12 | -0.45 | -0.42 | -0.80 | -0.87 | -0.96 |
| **71** | -0.16 | -0.45 | -0.29 | -0.69 | -0.92 | -0.94 |
| **72** | -0.30 | -0.38 | -0.37 | -0.81 | -0.94 | -0.95 |
| **73** | -0.33 | -0.41 | -0.33 | -0.85 | -0.91 | -0.96 |
| **74** | -0.31 | -0.44 | -0.38 | -0.86 | -0.89 | -0.94 |
| **75** | -0.31 | -0.43 | -0.35 | -0.86 | -0.85 | -0.92 |
| **76** | -0.31 | -0.39 | -0.31 | -0.92 | -0.87 | -0.96 |
| **77** | -0.20 | -0.43 | -0.21 | -0.79 | -0.94 | -0.94 |
| **78** | -0.27 | -0.43 | -0.34 | -0.79 | -0.90 | -0.95 |
| **79** | -0.27 | -0.41 | -0.35 | -0.78 | -0.89 | -0.92 |
| **80** | -0.24 | -0.41 | -0.31 | -0.75 | -0.83 | -0.89 |
| **81** [b] | -0.16 | -0.35 | -0.34 | -0.62 | -0.80 | -0.82 |
| **82** [b] | -0.20 | -0.37 | -0.31 | -0.67 | -0.79 | -0.85 |
| **83** [b] | -0.19 | -0.42 | -0.37 | -0.69 | -0.78 | -0.83 |
| **84** [b] | -0.18 | -0.40 | -0.37 | -0.68 | -0.78 | -0.82 |
| **85** [b] | -0.20 | -0.35 | -0.31 | -0.75 | -0.76 | -0.86 |
| **86** | -0.17 | -0.38 | -0.34 | -0.66 | -0.72 | -0.78 |
| **87** | -0.21 | -0.40 | -0.38 | -0.65 | -0.67 | -0.76 |
| **88** | -0.20 | -0.39 | -0.34 | -0.69 | -0.74 | -0.82 |
| **89** [b] | -0.07 | -0.39 | -0.20 | -0.61 | -0.81 | -0.82 |
| **90** [b] | -0.15 | -0.40 | -0.33 | -0.61 | -0.78 | -0.84 |
| **91** [b] | -0.14 | -0.37 | -0.34 | -0.60 | -0.77 | -0.81 |
| **92** [b] | -0.13 | -0.38 | -0.31 | -0.58 | -0.73 | -0.79 |
| **93** | -0.27 | -0.41 | -0.44 | -0.60 | -0.77 | -0.81 |
| **94** | -0.32 | -0.43 | -0.42 | -0.64 | -0.78 | -0.85 |
| **95** | -0.30 | -0.45 | -0.47 | -0.65 | -0.77 | -0.84 |
| **96** | -0.32 | -0.41 | -0.42 | -0.71 | -0.76 | -0.88 |
| **97** | -0.32 | -0.45 | -0.45 | -0.66 | -0.75 | -0.85 |
| **98** | -0.20 | -0.45 | -0.32 | -0.58 | -0.80 | -0.84 |
| **99** | -0.27 | -0.45 | -0.44 | -0.58 | -0.77 | -0.85 |
| **100** | -0.26 | -0.43 | -0.44 | -0.58 | -0.76 | -0.82 |
| **101** | -0.25 | -0.43 | -0.42 | -0.56 | -0.73 | -0.82 |
| **102** | -0.20 | -0.32 | -0.41 | -0.72 | -0.72 | -0.71 |
| **103** | -0.20 | -0.41 | -0.43 | -0.74 | -0.73 | -0.74 |
| **104** | -0.18 | -0.35 | -0.43 | -0.69 | -0.72 | -0.71 |
| **105** | -0.19 | -0.35 | -0.41 | -0.70 | -0.71 | -0.71 |
| **106** | -0.23 | -0.37 | -0.44 | -0.77 | -0.72 | -0.72 |
| **107** | -0.23 | -0.36 | -0.42 | -0.74 | -0.69 | -0.69 |
| **108** | -0.21 | -0.36 | -0.42 | -0.74 | -0.68 | -0.68 |
| **109** | -0.24 | -0.46 | -0.45 | -0.78 | -0.74 | -0.77 |
| **110** | -0.22 | -0.39 | -0.45 | -0.74 | -0.72 | -0.73 |

| | | | | | | |
|---|---|---|---|---|---|---|
| **111** | -0.23 | -0.39 | -0.44 | -0.75 | -0.71 | -0.73 |
| **112** | -0.26 | -0.38 | -0.46 | -0.73 | -0.63 | -0.69 |
| **113** | -0.29 | -0.41 | -0.48 | -0.77 | -0.64 | -0.72 |
| **114** | -0.26 | -0.45 | -0.47 | -0.75 | -0.65 | -0.74 |
| **115** | -0.24 | -0.39 | -0.47 | -0.70 | -0.64 | -0.70 |
| **116** | -0.25 | -0.39 | -0.46 | -0.71 | -0.63 | -0.71 |
| **117** | -0.26 | -0.43 | -0.40 | -0.77 | -0.75 | -0.79 |
| **118** | -0.19 | -0.35 | -0.41 | -0.70 | -0.71 | -0.71 |
| **119** | -0.19 | -0.40 | -0.42 | -0.71 | -0.71 | -0.71 |
| **120** | -0.17 | -0.33 | -0.41 | -0.66 | -0.69 | -0.67 |
| **121** | -0.18 | -0.33 | -0.40 | -0.67 | -0.68 | -0.67 |
| **122** | -0.16 | -0.24 | -0.31 | -0.62 | -0.62 | -0.64 |
| **123** | -0.20 | -0.29 | -0.34 | -0.67 | -0.63 | -0.67 |
| **124** | -0.10 | -0.28 | -0.20 | -0.60 | -0.65 | -0.67 |
| **125** | -0.16 | -0.26 | -0.32 | -0.60 | -0.62 | -0.65 |
| **126** | -0.15 | -0.27 | -0.30 | -0.58 | -0.60 | -0.66 |

GPCR – G-protein-coupled receptor. a – data from Ref. [20]; b – data from Ref. [30]. Interpretation of the bioactivity score: >0.00: active; −5.00−0.00: moderately active; <−5.00: inactive [36-38].

*2. Establishment of the QSAR model*

The possible QSAR models (Table S2) to predict BBB permeability were derived taking into account possible combinations of descriptors, *i.e.* lipophilicity (log $k_{w,\ IAM}$), molecular size (MW or α), electron properties (TPSA or HBD+HBA) and flexibility (NRB), similar to those described in our earlier studies [39].

**Table S2.** Statistics of the derived QSAR models.

| **No.** | **Model** | **$R^2$** | **$R^2_{adj}$** | **$R^2_{pred}$** | **PRESS** | **VIF** |
|---|---|---|---|---|---|---|
| M1 | log BB vs. (log $k_{w,\ IAM}$, MW, TPSA, NRB) | 0.7979 | 0.7912 | 0.7779 | 5.57810 | <3.0 |
| M2 | log BB vs. (log $k_{w,\ IAM}$, α, TPSA, NRB) | 0.8532 | 0.8483 | 0.8390 | 4.04364 | <2.8 |
| M3 | log BB vs. (log $k_{w,\ IAM}$, MW, HBD+HBA, NRB) | 0.9344 | 0.9323 | 0.9291 | 1.78148 | <2.9 |
| M4 | log BB vs. (log $k_{w,\ IAM}$, α, HBD+HBA, NRB) | 0.9610 | 0.9597 | 0.9576 | 1.06428 | <2.9 |
| M5 | log BB vs. (log $k_{w,\ IAM}$, MW, TPSA) | 0.7845 | 0.7792 | 0.7683 | 5.81996 | <2.4 |
| M6 | log BB vs. (log $k_{w,\ IAM}$, α, TPSA) | 0.8241 | 0.8197 | 0.8116 | 4.73318 | <2.4 |
| M7 | log BB vs. (log $k_{w,\ IAM}$, MW, HBD+HBA) | 0.9342 | 0.9326 | 0.9296 | 1.76752 | <2.8 |
| M8 | log BB vs. (log $k_{w,\ IAM}$, α, HBD+HBA) | 0.9579 | 0.9568 | 0.9547 | 1.13883 | <2.6 |

Two of the analyzed QSAR models show the best statistics, *i.e.* the M7 and M8 models. Both are very good and highly predictive. For further research, we have chosen the M7 model because it allows predicting the log BB from easily available data, *i.e.,* MW values and a total content of HBD+HBA. The polarizability of a molecule (α) can also be calculated on the basis of its structure, but it requires an expensive software, not available in every laboratory. The comparison of models M3 with M7 and M4 with M8 indicates that the flexibility of the molecule, described by the NRB value, has a little effect on the blood-brain barrier permeability. By limiting the number of independent variables, we do not take this parameter into account in the QSAR equations.

## References


1. Asif, M. Pharmacological activities of Triazole analogues as antibacterial, antifungal, antiviral agents. *Pharm. Sci. Asia* **2017**, *44*, 59-74.
2. Tzvetkov, N.T.; Euler, H.; Müller, C.E. Regioselective synthesis of 7,8-dihydroimidazo[5,1-*c*][1,2,4]triazine-3,6(2*H*,4*H*)-dione derivatives: A new drug-like heterocyclic scaffold. *Beilstein J. Org. Chem.* **2012**, *8*, 1584-1593.
3. Sztanke, K.; Tuzimski, T.; Rzymowska, J.; Pasternak, K.; Kandefer-Szerszeń, M. Synthesis, determination of the lipophilicity, anticancer and antimicrobial properties of some fused 1,2,4-triazole derivatives. *Eur. J. Med. Chem.* **2008**, *43*, 404–419.
4. Sztanke, M.; Rzymowska, J.; Janicka, M.; Sztanke, K. Two novel classes of fused azaisocytosine-containing congeners as promising drug candidates: Design, synthesis as well as *in vitro*, *ex vivo* and *in silico* studies. *Bioorg. Chem.* **2020**, *95*, 103480.
5. Mazur, L.; Łyszczek, R.; Sztanke, M.; Sztanke, K. Crystal structure of 8-(4-methylphenyl)-3-(propan-2-yl)-7,8-dihydroimidazo[2,1-c][1,2,4]triazin-4(6*H*)-one: A potential antitumor agent. *Acta Pol. Pharm.-Drug Res.* **2022**, *79*, 283–288. [
6. Sztanke, M.; Rzymowska, J.; Janicka, M.; Sztanke, K. Synthesis, structure confirmation, identification of *in vitro* antiproliferative activities and correlation of determined lipophilicity parameters with *in silico* bioactivity descriptors of two novel classes of fused azaisocytosine-like congeners. *Arabian J. Chem.* **2019**, *12*, 5302–5324.
7. Sztanke, K.; Rzymowska, J.; Niemczyk, M.; Dybała, I.; Kozioł, A.E. Synthesis, crystal structure and anticancer activity of novel derivatives of ethyl 1-(4-oxo-8-aryl-4,6,7,8-tetrahydroimidazo[2,1-*c*][1,2,4]triazin-3-yl)formate. *Eur. J. Med. Chem.* **2006**, *41*, 539–547.
8. Sztanke, K.; Tkaczyński, T. Synthesis of new derivatives of methyl 8-phenyl-6,7-dihydro-4-oxoimidazo[2,1-*c*][1,2,4]triazine-3-acetate. *Acta Pol. Pharm.-Drug Res.* **1997**, *54*, 147–149.
9. Sztanke, M.; Rzymowska, J.; Sztanke, K. Synthesis, structure elucidation and *in vitro* anticancer activities of novel derivatives of diethyl (2E)-2-[(2E)-(1-arylimidazolidin-2-ylidene)hydrazono]succinate and ethyl(4-oxo-8-aryl-4,6,7,8-tetrahydroimidazo[2,1-*c*][1,2,4]triazin-3-yl)acetate. *Bioorg. Med. Chem.* **2013**, *21*, 7465–7480.
10. Sztanke; K., Pasternak; K., Rzymowska; .J, Sztanke; M., Kandefer-Szerszeń; M. Synthesis, structure elucidation and identification of antitumoural properties of novel fused 1,2,4-triazine aryl derivatives. *Eur. J. Med. Chem.* **2008**, *43*, 1085–1094.
11. Sztanke, K.; Tkaczyński, T. Synthesis of new derivatives of 8-phenyl-7,8-dihydro-4-oxoimidazo[2,1-*c*][1,2,4]triazine-3-acetic acid hydrazide. *Acta Pol. Pharm.-Drug Res.* **1998**, *55*, 251–252.
12. Sztanke, K.; Tuzimski, T.; Sztanke, M.; Rzymowska, J.; Pasternak, K. Synthesis, structure elucidation, determination of the lipophilicity and identification of antitumour activities *in vitro* of novel 3-(2-furanyl)-8-aryl-7,8-dihydroimidazo[2,1-*c*][1,2,4]triazin-4(6*H*)-ones with a low cytotoxicity towards normal human skin fibroblast cells. *Bioorg. Med. Chem.* **2011**, *19*, 5103–5116.
13. Sztanke, M.; Rzymowska, J.; Sztanke, K. Synthesis, structure elucidation and identification of antiproliferative activities of a novel class of thiophene bioisosteres bearing the privileged 7,8-dihydroimidazo[2,1-*c*][1,2,4]triazin-4(6*H*)-one scaffold. *Bioorg. Med. Chem.* **2015**, *23*, 3448–3456.
14. Sztanke, K.; Pasternak, K.; Sztanke, M.; Kandefer-Szerszeń, M.; Kozioł, A.E.; Dybała, I. Crystal structure, antitumour and antimetastatic activities of disubstituted fused 1,2,4-triazinones. *Bioorg. Med. Chem. Lett.* **2009**, *19*, 5095–5100.
15. Sztanke, M.; Rzymowska J., Janicka, M., Sztanke, K. Synthesis, structure elucidation, determination of antiproliferative activities, lipophilicity indices and pharmacokinetic properties of novel fused azaisocytosine-containing congeners. *Arabian J. Chem.* **2019**, *12*, 4044–4064.
16. Sztanke, K.; Tkaczyński, T. Synthesis of new derivatives of 3-benzyl-8-aryl-7,8-dihydro-6*H*-imidazo[2,1-*c*][1,2,4]triazin-4-one. *Acta Pol. Pharm.-Drug Res.* **1997**, *54*, 71–74.
17. Hordyjewska, A.; Sztanke, K.; Radej, S.; Rzymowska, J.; Pasternak, K.; Roliński, J. Antiproliferative activity of new synthesized derivatives of imidazotriazinone in breast cancer cell line (T47D). In: *Pierwiastki, środowisko i życie człowieka;* Pasternak K. (Ed.); Pol. Tow. Magnezol.: Lublin, Poland, 2009, pp. 75–79.
18. Sztanke, M.; Rzymowska, J.; Sztanke K. Anticancer active trifluoromethylated fused triazinones are safe for early-life stages of zebrafish (*Danio rerio*) and reveal a proapoptotic action. *J. Enzyme Inhib. Med. Chem.* **2021**, *6*, 335–343.
19. Sztanke, M.; Sztanke, K.; Rajtar, B.; Świątek, Ł.; Boguszewska, A.; Polz-Dacewicz, M. The influence of some promising fused azaisocytosine-containing congeners on zebrafish (*Danio rerio*) embryos/larvae and their antihaemolytic, antitumour and antiviral activities. *Eur. J. Pharm. Sci.* **2019**, *132*, 34–43.
20. Sztanke, M.; Rzymowska, J.; Sztanke, K. Anti-tumor active isopropylated fused azaisocytosine-containing congeners are safe for developing *Danio rerio* as well as red blood cells and activate apoptotic caspases in human breast carcinoma cells. *Molecules* **2022**, *27*, 1211.
21. Sztanke, M.; Sztanke, K. Biologically important hydrazide-containing fused azaisocytosines as antioxidant agents. *Redox Rep.* **2017**, *22*, 572–581.
22. Szuster-Ciesielska, A.; Sztanke, K.; Kandefer-Szerszeń, M. A novel fused 1,2,4-triazine aryl derivative as antioxidant and non-selective antagonist of adenosine $A_{2A}$ receptors in ethanol-activated liver stellate cells. *Chem. Biol. Interact.* **2012**, *195*, 18–24.
23. Worzakowska, M.; Sztanke, M.; Sztanke K. Thermal properties and decomposition mechanism of disubstituted fused 1,2,4-triazoles considered as potential anticancer and antibacterial agents. *J. Therm. Anal. Calorim.* **2022**, *147*, 14315–14327.
24. Worzakowska, M.; Sztanke, M.; Sztanke, K. Pyrolysis and oxidative decomposition mechanism of trifluoromethylated fused triazinones. *J. Anal. Appl. Pyrolysis* **2021**, *157*, 105226.

25. Worzakowska, M.; Sztanke, M.; Sztanke, K. Experimental Studies on the Thermal Properties and Decomposition Course of a Novel Class of Heterocyclic Anticancer Drug Candidates. *Int. J. Mol. Sci.* **2023**, *24*, 6190.
26. Ostasz, A.; Łyszczek, R.; Sztanke, K.; Sztanke, M. TG-DSC and TG-FTIR Studies of Annelated Triazinylacetic Acid Ethyl Esters – Potential Anticancer Agents. *Molecules* **2023**, *28*, 1735.
27. Bartyzel, A.; Sztanke, M.; Sztanke, K. An insight into the thermal behaviour of biologically active 8-aryl-4-oxo-4,6,7,8-tetrahydroimidazo[2,1-*c*][1,2,4]triazine-3-carbohydrazides. *J. Anal. Appl. Pyrolysis* **2016**, *121*, 138–145.
28. Bartyzel, A.; Sztanke, M.; Sztanke, K. Thermal behaviour of antiproliferative active 3-(2-furanyl)-8-aryl-7,8-dihydroimidazo[2,1-*c*][1,2,4]triazin-4(6*H*)-ones. *J. Therm. Anal. Calorim.* **2017**, *130*, 1541–1551.
29. Łyszczek, R.; Bartyzel, A.; Głuchowska, H.; Mazur, L.; Sztanke, M.; Sztanke, K. Thermal investigations of biologically important fused azaisocytosine-containing congeners and the crystal structure of one representative. *J. Anal. Appl. Pyrolysis* **2018**, *155*, 141–151.
30. Sztanke, M.; Sztanke, K.; Ostasz, A.; Głuchowska, H.; Łyszczek, R. Thermal Investigations of Annelated Triazinones—Potential Analgesic and Anticancer Agents. *Molecules* **2023**, *28*, 6542.
31. Worzakowska, M.; Sztanke, M.; Sztanke, K. Decomposition mechanism of annelated triazinones bearing the *para*-nitrophenyl group. *J. Anal. Appl. Pyrolysis* **2020**, *149*, 104856.
32. Tyszczuk-Rotko, K.; Sztanke, M.; Sasal, A.; Sztanke, K. Voltammetry as the First Method for Direct Determination of a Novel Antagonist of $A_{2A}$ Adenosine Receptors. *Electroanalysis* **2019**, *31*, 2480–2487.
33. Stępniowska, A.; Sztanke, M.; Tuzimski, T., Korolczuk, M.; Sztanke, K. A simple stripping voltammetric method for the determination of a new anticancer prodrug in serum. *Biosens. Bioelectron*. **2017**, *94*, 584–588.
34. Kozak, J.; Tyszczuk-Rotko, K.; Sadok, I.; Sztanke, K.; Sztanke, M. Application of screen-printed sensor modified with carbon nanofibers for the voltammetric analysis of an anticancer disubstituted fused triazinone. *Int. J. Mol. Sci.* **2022**, *23*, 2429.
35. Kozak, J.; Tyszczuk-Rotko, K.; Sztanke, K.; Sztanke, M. Sensitive and Selective Voltammetric Sensor Based on Anionic Surfactant-Modified Screen-Printed Carbon for the Quantitative Analysis of an Anticancer Active Fused Azaisocytosine-Containing Congener. *Int. J. Mol. Sci.* **2023**, *24*, 564.
36. Jeelani, A.; Muthu, S.; Narayana, B. Molecular structure determination, bioactivity score, spectroscopic and quantum computational studies on (E)-N′-(4-chlorobenzylidene)-2-(napthalen-2-yloxy) acetohydrazide. *J. Mol. Struct.* **2021**, *1241*, 130558.
37. Hussein, Y.T.; Azeez, Y.H. DFT analysis and in silico exploration of drug-likeness, toxicity prediction, bioactivity score, and chemical reactivity properties of the urolithins. *J. Biomol. Struct. Dyn.* **2023**, *41*, 1168–1177.
38. Flores-Holguín, N.; Frau, J.; Glossman-Mitnik, D. Chemical Reactivity Properties and Bioactivity Scores of the Angiotensin II Vasoconstrictor Octapeptide. In Cheminformatics and Its Applications; IntechOpen: London, UK, 2020.
39. Janicka, M.; Sliwińska, A.; Sztanke, M.; Sztanke, K. Combined Micellar Liquid Chromatography Technique and QSARs Modeling in Predicting the Blood–Brain Barrier Permeation of Heterocyclic Drug-like Compounds. *Int. J. Mol. Sci*. **2022**, 23, 15887.
